# Pelagic tunicates at shallow hydrothermal vents of Kueishantao

**DOI:** 10.1371/journal.pone.0225387

**Published:** 2019-12-05

**Authors:** Pietro Franco, Hans-Uwe Dahms, Jiang-Shiou Hwang

**Affiliations:** 1 Institute of Marine Biology, National Taiwan Ocean University, Keelung, Taiwan, R.O.C; 2 Department of Biomedical Science and Environmental Biology, Kaohsiung Medical University, Kaoshiung, Taiwan; 3 Department of Marine Biotechnology and Resources, National Sun Yat-sen University, Kaohsiung, Taiwan; 4 Center of Excellence for the Oceans, National Taiwan Ocean University, Keelung, Taiwan; Zhejiang University College of Life Sciences, CHINA

## Abstract

The composition and abundance of the major zooplankton taxon tunicates were analyzed in a multi-year study and correlated with environmental parameters in the area around the hydrothermal vent field of Kueishantao (Turtle Island) in Taiwan. This provided the first study about tunicates above hydrothermal vents worldwide. We chose seven different stations for sample collection. Sampling was carried out in September of 2009, 2014, and 2015 (autumn) and June 2015 (summer). A total of ten tunicate species were identified belonging to the classes Appendicularia and Thaliacea during the above periods throughout the area. Considering the limited diversity of these organisms worldwide (40 species are identified in each class), we affirm that Kueishantao, a shallow water hydrothermal vent field, with 10 species provided an unexpected biodiversity hot spot for pelagic tunicates. The sampling of the organisms in the surface waters provided higher abundances compared to oblique tows. Comparing results from three autumn seasons we found that temperature values correlated with changes in tunicate abundances. We discovered strong seasonal changes in pelagic tunicate abundances over the entire survey period, with the highest abundances observed during autumn.

## Introduction

Pelagic tunicates are present in marine waters worldwide. Scientists are increasingly interested in their ecological roles in marine ecosystem [1,2,3]. Their reproductive cycles and life histories are adapted to different environmental conditions [4]. Bacteria and phytoplankton represent their staple food and are secured through suspension feeding [5]. Pelagic tunicates may play an important link for carbon flow from the microbial to the meso- and macrozooplankton loop [6].

The distribution of pelagic tunicates is affected by seasonal changes in water movement and their associated temperature and salinity structures [7]. Research on pelagic tunicates in the waters of the China Seas is limited [8,9]. In northern Taiwan there are only a few studies on pelagic tunicates [10,11].

However, little is known about the ecology of pelagic tunicates adjacent to hydrothermal vent waters. Kueishantao is situated in the northeastern part of Taiwan and belongs to Yilan County. Kueishantao is an active volcano with the presence of active hydrothermal vents. The Kueishantao hydrothermal vent field is about 0.5 km^2^) is situated in shallow waters southeast of Kueishantao. The area surrounding the field is characterized by a seafloor with lava and pyroclastic sediments. There are several hydrothermal vents in shallower waters (15–300 m depth) with the lowest recorded vent water pH worldwide [12].

Gases produced here at the vent sites are mainly composed of carbon dioxide and a small amount of hydrogen sulfide. which later is assimilated to sulfur by sulfurbacteria that causes the light-blue coloration of the waters around Kueishantao. The vents can be divided into ‘‘yellow spring” and ‘‘white spring” types. The temperature of the yellow-spring fluids is between 78 and 116°C, and the temperatures of the white-spring fluids are between 30°C and 65°C). Yellow-spring effluents have a very low pH (as low as 1.52) and a wide range of chemical compositions. White-spring effluents are characterized by relatively low concentrations of copper, iron, and methane The effluents from the vents mostly contain hydrogensulfite. Like in most shallow-water vent ecosystems energy is supplied here by both photosynthesis and chemosynthesis [13].

This study was divided in three different approaches. The first approach compared surface collection and oblique tow sampling of pelagic tunicates during autumn (October 2015). Our hypothesis is that the two techniques provide different results in terms of occurrence and abundance of pelagic tunicates. In a second approach abundances and distribution of pelagic tunicates were compared throughout a period of three autumns (2009, 2014, 2015), assuming interannual differences for the area and studied seasons. Following a third hypothesis that seasonal differences occur, a seasonal comparison between June (summer) and October (autumn) of 2015 was done to understand seasonal differences in species diversity and abundances of pelagic tunicates.

## Material and methods

There was no need for any permit. We got research support for this research at Kuaishantao from the Ministry of Science and Technology (MOST, Taiwan). We confirm that the field studies did not include endangered or protected species.

**Fig 1:**
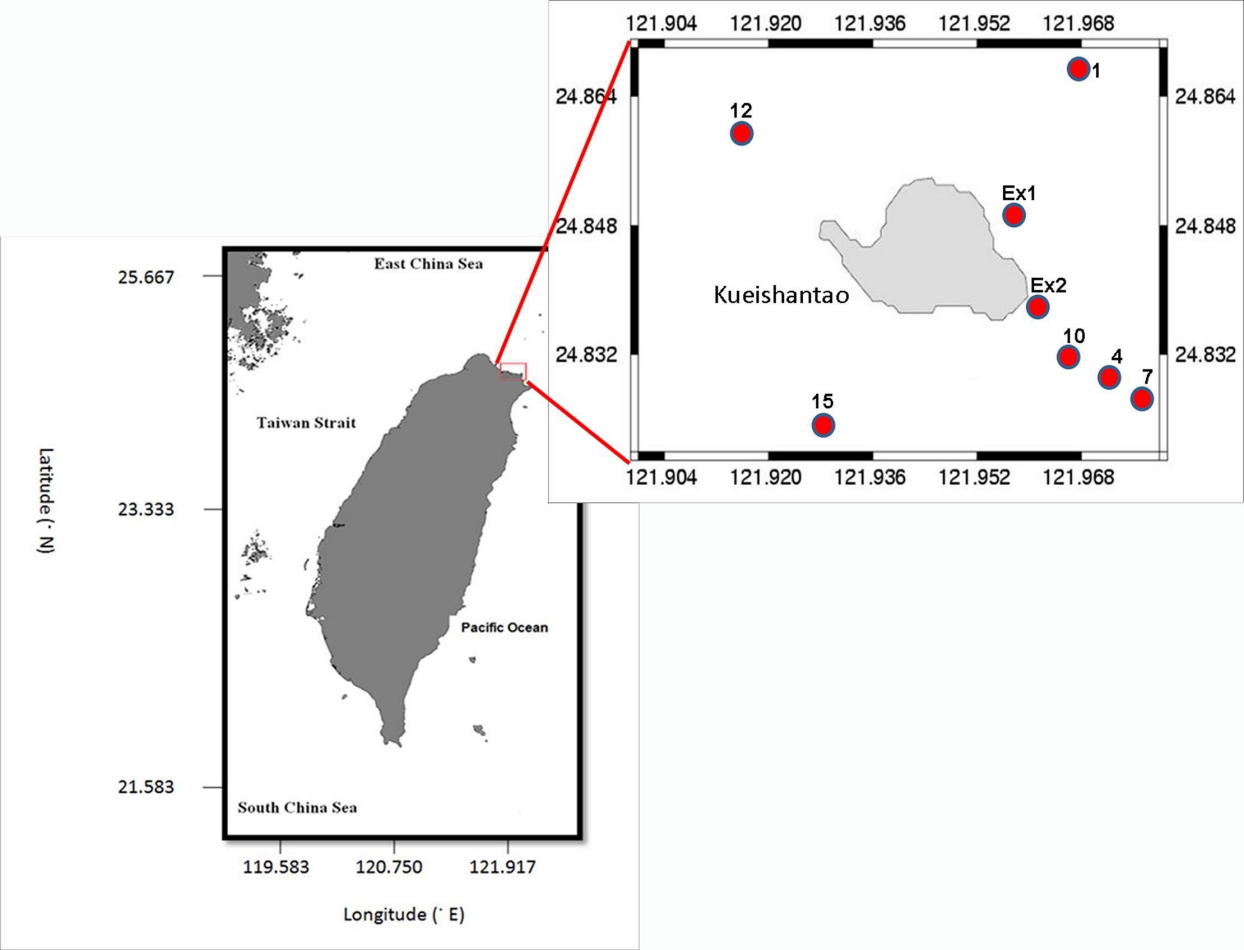
Location of sampling stations in the waters around Turtle Island in Taiwan.

Zooplankton samples were collected from the surface waters around Kueishantao (Turtle Island), in NE Taiwan. We used a 200 μm mesh size zooplankton net (180 cm long, 45 cm mouth diameter). We chose eight different stations for the collection of mesozooplankton (**Fig 1**). The sampling was carried out in September 2009 (autumn), September 2014 (autumn), October 2015 (autumn), and June 2015 (summer). Finally, we collected pelagic tunicates from seven stations in 2015 (autumn), using an oblique tow. After collection, the samples were preserved in 5% buffered formalin. Later in the lab, identification and counting was done using a stereomicroscope. Identification aids were retrieved from the Marine Species Data Portal (http://species-identification.org/) and from the World Register of Marine Species (WoRMS, 2016 - http://www.marinespecies.org/.) Available from VLIZ (accessed 2016-04/05. doi:10.14284/170). The abundance was expressed as ind./m^3^. During the cruise we also collected environmental data, including temperature, and salinity, and chl-*a* obtained via a multi-parameter sensor (CTD).

All data mapping concerned with abundance and distribution was made using the software SURFER 8.0. As for statistical analysis for the second and third part of our study, the different parameters (temperature, salinity, *Oikopleura* and Thaliacea abundances) were tested throughout the different observation periods. We tested for normality and equal variance for the data set. One-way ANOVA was selected when these two criteria were met. ANOVA on ranks was selected when these two criteria were not met. For all tests, the significance level was p = 0.05. Finally, the variables that had any significant changes over time and season were tested for any correlative effect by the Pearson correlation model. The objective was to determine which environmental factors were most significantly affecting the abundance of tunicates (grouped in the two taxonomic classes: Larvacea and Thaliacea) in the areas around Turtle Island.

## Results

### Surface collection *vs*. oblique tow collection in autumn 2015

#### Taxonomic composition, spatial distribution, and abundance

A total of eight species were identified: *O*. *dioica*, *O*. *rufescens*, *O*. *longicauda*, *F*. *aberrans* belonging to the class Appendicularia and *D*. *denticulatum*, *D*. *gegenbauri*, *T*. *democratica orientalis*, *D*. *gegenbauri* belonging to the Thaliacea. When the Appendiculariidae and Fritillariidae could not be identified to species level, we refered to them as *Oikopleura* sp. and *Fritillaria* sp.

**Table 1** shows a list of the species encountered and their abundances during the survey for every station. For every species, the table shows the mean abundance in percentage (%) at the same station for the two different sampling techniques. The last row shows the total mean abundances for every zone expressed in ind./m^3^. The family Appendiculariidae, belonging to the class Larvacea was most abundant throughout the area, with the highest abundance values measured. *O*. *dioica* and *O*. *longicauda* were the dominant species.

**Table 1:** Relative abundance of the different species at the different sampling stations using two different collecting techniques.

Relative abundance (%)																
	Surface sampling								Oblique tow sampling							
Zooplankton	Ex1	Ex2	1	4	7	10	12	15	Ex1	Ex2	1	4	7	10	12	15
**Larvacea:** **Appendiculariidae**																
*O*. *dioica*	29.5	27.8	23	35.9	33	34.8	31.1	27.9	35.3	46.3	33.1	59.7	39.7	25.2	42	29.9
*O*. *longicauda*	13.5	15.9	15.2	26.9	24.8	26	19.7	17.1	31.5	15.5	13.3	26.8	19.6	33.3	23.1	15.3
*O*. *rufescens*	8.2	3.9	3.3	6.6	1.1	1	6.6	5.9	5.1	6	3.7	12.9	8.4	6	10.7	8.1
*Oikopleura* sp.	34.4	35.8	48	27.3	38.7	31.7	41.8	44.5	26.3	27.7	45	10.5	32.3	16.8	22.2	44.3
**Family: Fritillariidae**																
*F*. *aberrans*	-	-	5	-	-	-	-	1.9	-	-	2.3	1.5	-	-	-	1.5
*F*. *species*	14.2	16.7	5.5	3.3	2.4	6.5	0.8	1.2	1.8	3.9	2.6	1.1	-	2.7	2	1.4
**Total amount (Larvacea) (ind./m**^**3**^**)**	**82.9**	**97.2**	**570.3**	**57.6**	**137.1**	**82.1**	**2180.8**	**1224.8**	**367.8**	**137.9**	**240.6**	**34**	**134.3**	**122.3**	**184.5**	**135.4**
**Thaliacea:Doliolidae**																
*D*. *denticulatum*	-	-	86.2	41.3	-	-	-	27.2	-	73	68.2	2	68	51	-	-
*D*. *gegenbauri*	-	-	13.8	10.3	-	-	-	19.8	-	22	12.8	7.4	15.3	24	-	-
**Salpidae**																
*T*. *democratica*	88.4	86.6	-	44.7	67.9	66.6	83.1	23.4	100	5	19	90.6	16.7	25	100	100
*Brooksia rostrata*	11.6	13.4	-	3.7	32.7	33.3	16.9	29.8	-	-	-	-	-	-	-	-
**Total amount (Thaliacea) (ind./m**^**3**^**)**	**5.2**	**6**	**95.9**	**31.7**	**11.9**	**5.4**	**149.4**	**195.2**	**4.2**	**12.5**	**39.5**	**13.9**	**11.7**	**4.4**	**20.8**	**6.4**

**Fig 2:**
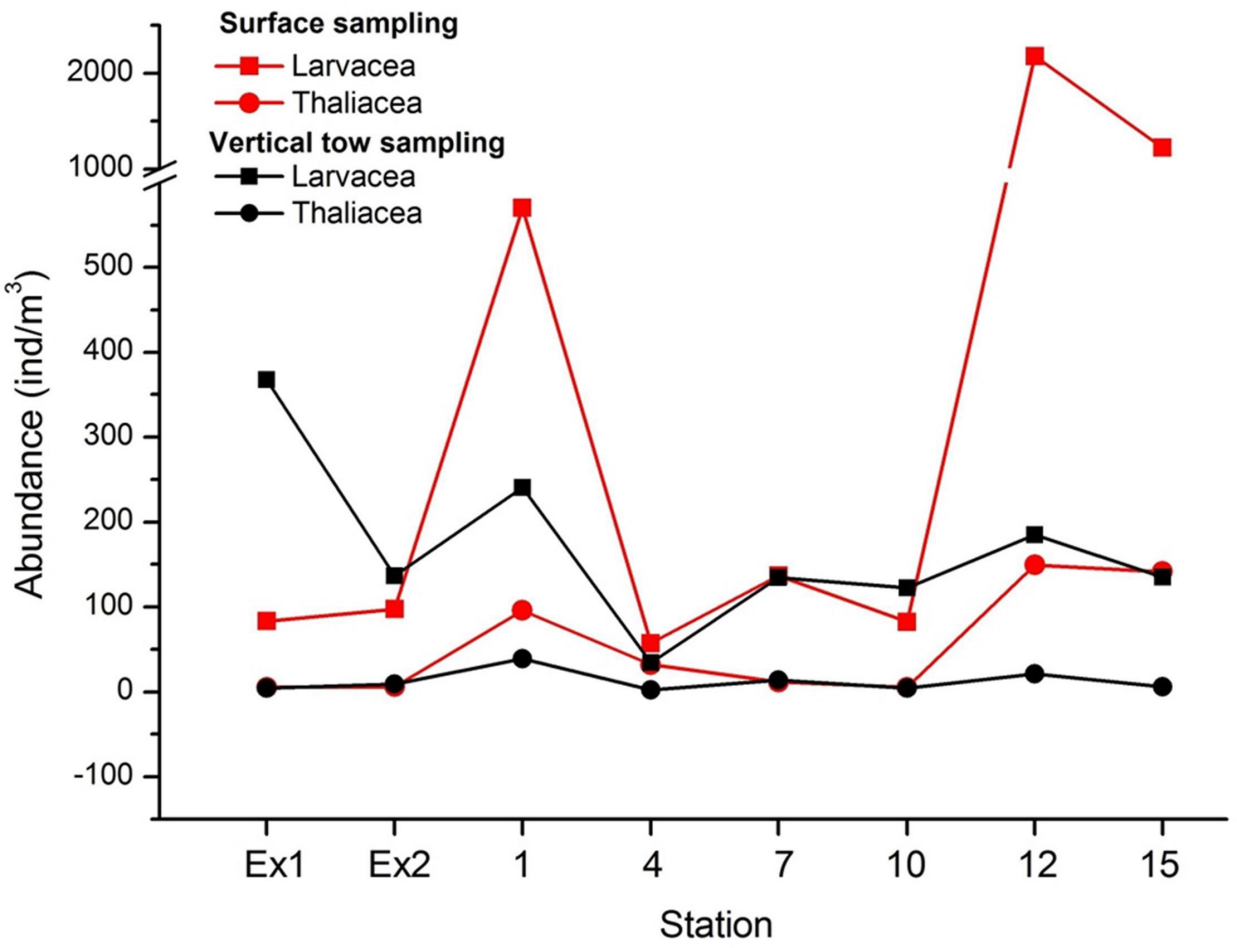
Abundance of larvaceans and thaliaceans at the sampling stations collected with two different sampling techniques (surface sampling *vs*. oblique tow sampling).

**Fig 2** shows the abundances of the two classes of organisms (Larvacea and Thaliacea) at the 8 stations collected with two different sampling techniques. The abundances of larvaceans were always higher than those of thaliaceans (up to and more than 2000 ind./m^3^) and the surface sampling showed higher values of collected organisms compared to the oblique tow samples. The highest individual counts were made at stations 1, 12, and 15.

### Annual comparison between abundances and distribution of pelagic tunicates among the autumn seasons of 2009, 2014, 2015

#### Environmental conditions

The variations of mean surface temperature and salinity in the studied area are shown in **Table 2**. The changes of salinity are limited at the different sampling stations during the three years of study, ranging from 33.1 to 34.2. The surface temperature varied during the three years. In fact, surveying all stations, we found that the warmest autumn was in 2014 with values reaching 28.6°C. The lowest values were measured in autumn 2009, with a minimum temperature of 24.8°C.

**Table 2:** Table showing the changes in temperature (°C) and salinity during autumn 2009, 2014 and 2015.

Sampling season						
	Autumn 2009		Autumn 2014		Autumn 2015	
Station	Temperature (°C)	Salinity	Temperature (°C)	Salinity	Temperature (°C)	Salinity
**Ex 1**	26.1	33.7	28.5	33.7	25.9	33.8
**Ex 2**	25.4	33.8	28.6	33.7	25.7	34.1
**1**	24.8	33.8	28.6	33.6	26.1	33.9
**4**	26.2	33.7	29.3	33.6	26.3	34.2
**7**	26.4	33.6	28.1	33.6	25.6	33.9
**10**	26.5	33.1	27.7	33.6	26.1	34.1
**12**	26.6	32.7	28.1	33.6	25.7	33.9
**15**	26.2	33.7	28.5	33.7	25.8	33.9

#### Taxonomic composition, spatial distribution and abundance

A total of ten species were identified: *O*. *dioica*, *O*. *caphocera*, *O*. *rufescens*, *O*. *longicauda*, *F*. *aberrans* belonging to the Appendicularia and *D*. *muelleri*, *D*. *denticulatum*, *T*. *democratica orientalis*, *D*. *gegenbauri* and *T*. *vagina* belonging to the Thaliacea. When the Appendiculariidae could not be identified to species level, we referred to them as *Oikopleura* sp. The same holded for the doliolids, we referred to as *Doliolum* sp.

**Table 3** shows a list of the species and their abundances encountered during the survey in the three autumns of 2009/2014/2015. It shows the relative abundance for every station during the three seasons. For every species, the table shows the relative mean abundance in percentage (%) at every station throughout the four seasons. The last row shows the total mean abundances for every zone expressed in ind./m^3^. The family Appendiculariidae, belonging to the class Larvacea, was most abundant throughout the area. *O*. *dioica* and *O*. *longicauda* were the dominant species. Several larvaceans could not be identified at species level and high percentages of *Oikopleura* sp. were present. The highest abundance values of pelagic tunicates were measured in autumn 2015, for both classes.

**Table 3:** Relative abundances of the different species at the different sampling stations during the autumn seasons of 3 years.

Relative abundance (%)																								
	Autumn (2009)								Autumn (2014)								Autumn (2015)							
Zooplankton	Ex1	Ex2	1	4	7	10	12	15	Ex1	Ex2	1	4	7	10	12	15	Ex1	Ex2	1	4	7	10	12	15
**Larvacea: Appendiculariidae**																								
*O*. *dioica*	56.6	54.9	44	50.1	40.3	52.3	47.5	50	38	28.6	34.6	25.8	40.9	30.2	16.3	32.4	29.5	27.8	23	35.9	33	34.8	31.1	27.9
*O*. *longicauda*	25.9	15.8	37.6	26.3	22.6	25.5	30.7	25.4	17.8	7.7	7.2	13.5	15.4	7.7	11.3	8.4	13.5	15.9	15.2	26.9	24.8	26	19.7	17.1
*O*. *rufuscens*	5.7	15.6	7.5	14.8	22	9.9	12.5	14.8	15.0	13.7	5.6	18.2	3.7	9.8	6.0	4.5	8.2	3.9	3.3	6.6	1.1	1	6.6	5.9
*Oikopleura* sp.	10.4	6.1	10.7	7.7	9.2	5.2	6.4	2.9	10.5	45.5	48.7	38.9	34.9	46.6	57.8	50.7	34.4	35.8	48	27.3	38.7	31.7	41.8	44.5
**Family: Fritillariidae**																								
*F*. *aberrans*	-	-	2	-	-	1.1	1.9	3.9	10.0	-	2.6	-	-	0.0	4.0	1.4	-	-	5	-	-	-	-	1.9
*Fritillaria* sp.	1.4	7.6	2	0.5	5.9	5	1	3	8.6	15.0	1.3	-	0.4	5.6	4.4	2.6	14.2	16.7	5.5	3.3	2.4	6.5	0.8	1.2
**Total amount (ind./m**^**3**^**)**	**76.3**	**144.3**	**209.9**	**474.5**	**60.1**	**225.9**	**358.5**	**271.4**	**15.7**	**8.6**	**63.4**	**8.8**	**8.9**	**11.9**	**12.4**	**45.5**	**82.9**	**97.2**	**570.3**	**57.6**	**137**	**82.1**	**2180.8**	**1224.8**
**Class: Thaliacea** **Family: Doliolidae**																								
*D*. *denticulatum*	57.4	44	28.0	27.7	22.8	39.7	61.5	25.3	22.0	16.7	11.8	35	-	17.4	15.5	8.8	-	-	86.2	41.3	-	-	-	27.2
*D*. *muelleri*	7.3	4.6	3.1	1.5	-	-	3.2	0.0	-	3.9	-	3	-	3.2	2.2	2.5	-	-	-	-	-	-	-	-
*D*. *gegenbauri*	24.6	11.9	11.9	8.9	12.7	20.4	14.9	19.4	14.5	8.6	8.3	10		12.9	4.9	5.4	-	-	13.8	10.3	-	-	-	19.8
*Doliolum* sp.	-	29	25.3	20.6	15.9	8.4	20.4	6.7	15.3	9.0	17.2	32	85.8	9.1	6.7	7.3								
**Family: Salpidae**																								
*T*. *democratica*	10.8	10.4	31.5	38.4	49.1	30	-	48.5	44.2	56.7	56.8	15	5.2	52.5	65.2	69.2	88.4	86.6	-	44.7	67.9	66.6	83.1	23.4
*Brooksia rostrata*	-	-	-	3	-	1.5	-	-	4.1	5.1	5.8	10	9	5.0	5.6	6.8	11.6	13.4	-	3.7	32.7	33.3	16.9	29.8
*Thetys vagina*	-	-	-	-	-	-	-	-	-	-	0.2	-	-	-	-	-	-	-	-	-	-	-	-	-
**Total amount (ind./m**^**3**^**)**	**13.1**	**29.2**	**39.1**	**120.9**	**17.4**	**72.3**	**78.6**	**54.1**	**54.8**	**29.2**	**37.4**	**6.8**	**4.2**	**30.3**	**22.3**	**24.8**	**5.2**	**6**	**95.9**	**31.7**	**11.9**	**5.4**	**149.4**	**195.2**

#### Pelagic tunicates and environmental variables

**Fig 3:**
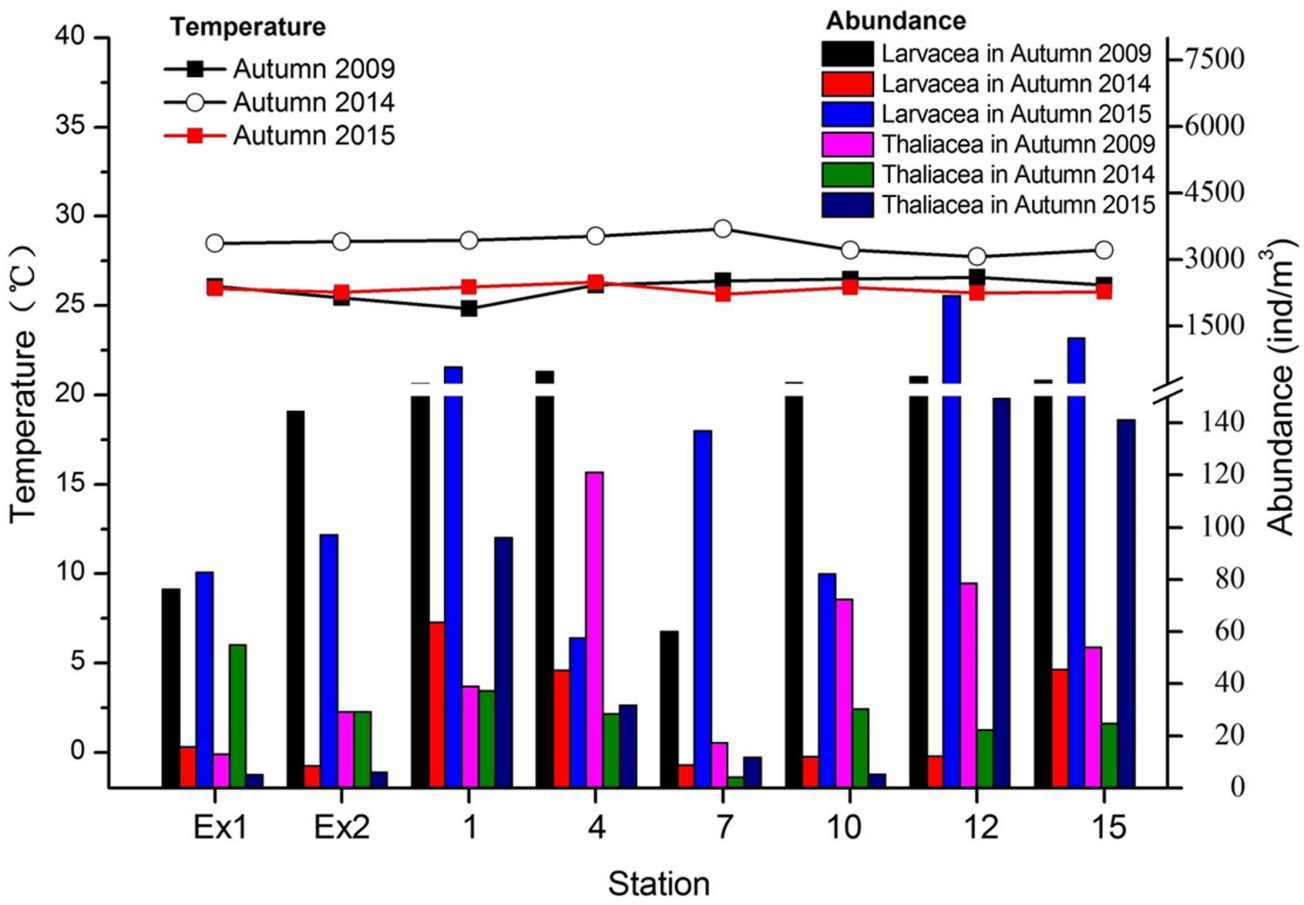
Total abundance of pelagic tunicates and the temperature values at each station during the three seasons of this study (autumn 2009/2014/2015).

**Fig 3** shows the total abundances of pelagic tunicates and temperature values in the study area during the autumns of three years. The figure shows mean values for every collection station. The highest concentrations of these organisms were measured during autumn 2015. Temperature showed the lowest values in 2014, when the lowest numbers of organisms were measured.

We used Pearson correlation to understand the environmental factors on the abundances of pelagic tunicates and the results are provided in **Table 4**. Only Larvaceans and Thaliaceans were positively correlated with each other. No correlation was found with environmental factors.

**Table 4:** Results of the Pearson correlation model analyzing the abundances of the two classes of organisms and environmental factors.

* *	Temperature	Salinity	Oikopleuridae	Thaliacea
**Temperature**	1			
**Salinity**	-0.30161	1		
**Oikopleuridae**	-0.3644	0.118433	1	
**Thaliacea**	-0.24584	-0.11082	0.833147	1

**Fig 4:**
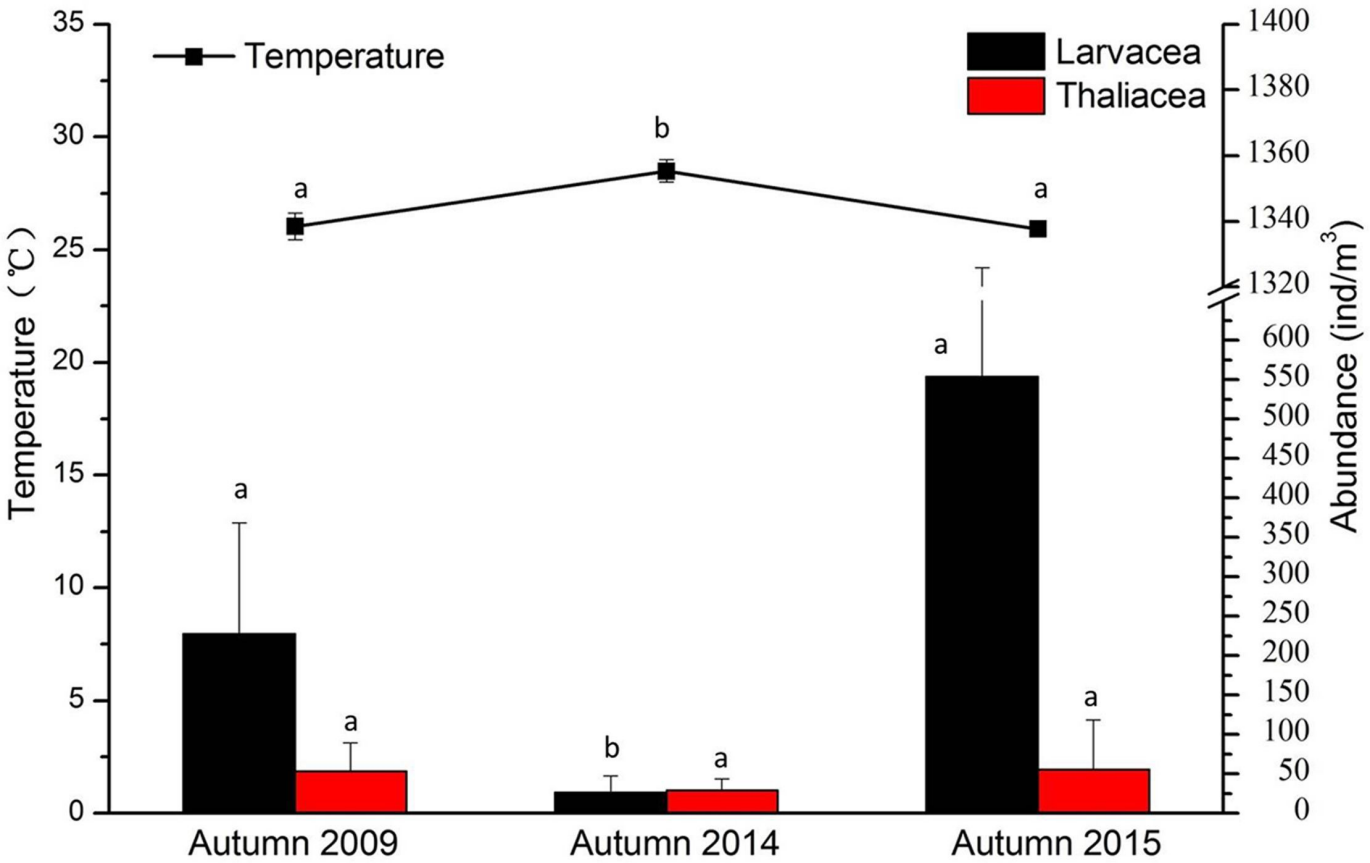
Changes in temperature and abundance of thaliaceans and Oikopleuridae for the three autumn seasons surveyed (average values for seasons and the entire area). Different letters indicate significant differences (p<0.05).

**Fig 4** shows the results of our statistical ANOVA model. The temperature decreased in 2014 where the model showed significant differences. The abundance of Larvacea dropped that year which was also indicated by the statistical model. The autumns of 2009 and 2015 did not show significant differences in terms of abundances.

### Comparison between abundances and distribution of organisms collected in summer 2015 and autumn 2015

#### Environmental conditions

The variations of mean surface temperature and salinity in the study area are shown in **Table 5**. The changes of salinity were limited to the different sampling stations during the period of three years, ranging from 33.1 to 34.1. As for the surface temperature, it varied during the two different seasons. Summer had a temperature averaging 29.3°C whereas autumn averaged at 26°C.

**Table 5:** Table showing the changes in temperature (°C) and salinity during summer 2015 and October 2015.

Sampling season				
	Summer 2015		Autumn 2015	
Station	Temperature (°C)	Salinity	Temperature (°C)	Salinity
**Ex 1**	29.3	33.9	25.9	33.8
**Ex 2**	29.4	33.9	25.7	34.1
**1**	29.3	34.1	26.1	33.8
**4**	29.5	34.3	26.3	34.2
**7**	29.2	33.9	25.6	33.9
**10**	28.4	34.1	26.1	34.1
**12**	29.6	34.1	25.7	33.9
**15**	29.3	33.9	25.8	33.9

### Taxonomic composition, spatial distribution and abundance

A total of nine species were identified: *O*. *dioica*, *O*. *caphocera*, *O*. *rufescens*, *O*. *longicauda*, *F*. *aberrans* belonging to the class Appendicularia; and *D*. *denticulatum*, *T*. *democratica orientalis*, *D*. *gegenbauri*, and *T*. *vagina* belonging to the Thaliacea.

**Table 6** shows a list of the species encountered during this survey in summer and autumn 2015 and their abundances in the study area. It shows the relative abundance for every station during the two seasons. For every species, the table shows the relative mean abundance in percentage (%) at every area throughout the four seasons. The last row shows the total mean abundances for every area expressed in ind./m^3^. The family Appendiculariidae, belonging to the class Larvacea, was most abundant throughout the area. *O*. *dioica* and *O*. *longicauda* were dominant species. Several larvaceans could not be identified at species level and high percentages of *Oikopleura* sp. were present. The highest abundance values for pelagic tunicates were measured in summer 2015, for both classes of organisms.

**Table 6:** Distribution of Oikopleuridae and Thaliacea in the study area in the two seasons (summer and autumn 2015).

Relative abundance (%)																
	Summer								Autumn							
Zooplankton	Ex1	Ex2	1	4	7	10	12	15	Ex1	Ex2	1	4	7	10	12	15
**Larvacea:** **Appendiculariidae**																
*O*. *dioica*	29.4	28.0	25.7	29	16.2	17.5	15.3	23.1	29.5	27.8	23	35.9	33	34.8	31.1	27.9
*O*. *longicauda*	22.0	13.7	15.0	21	13.2	21.5	19.5	14.4	13.5	15.9	15.2	26.9	24.8	26	19.7	17.1
*O*. *rufuscens*	6.2	4.3	9.2	6	8.8	6.3	7.8	8.3	8.2	3.9	3.3	6.6	1.1	1	6.6	5.9
*Oikopleura* sp.	38.7	51.6	50.1	36	57.4	52.3	50.8	52.0	34.4	35.8	48	27.3	38.7	31.7	41.8	44.5
**Family: Fritillariidae**								1.0								
*F*. *aberrans*	2.3	0.7	-	4	1.5	0.7	4.7	1.3	-	-	5	-	-	-	-	1.9
*Fritillaria sp*.	1.4	1.7	-	4	2.9	1.8	2.0	23.1	14.2	16.7	5.5	3.3	2.4	6.5	0.8	1.2
**Total amount (ind./m**^**3**^**)**	**146.6**	**257**	**180.3**	**146.2**	**266.5**	**142.3**	**141.5**	**335**	**82.9**	**97.2**	**570.3**	**57.6**	**137.1**	**82.1**	**2180.8**	**1224.8**
**Thaliacea:** **Doliolidae**																
*D*. *denticulatum*	48.5	56.8	-	-	15.8	33.3	38.3	27.7	-	-	86.2	41.3	-	-	-	27.2
*D*. *muelleri*	2	4.3	-	-	5	1.5	4.7	7.1								
*D*. *gegenbauri*	46.5	0.1	-	-	12.6	21.5	21.9	13.8	-	-	13.8	10.3	-	-	-	19.8
*Doliolum* sp.	3	38.9	-	-	9.8	37.9	35.2	19.1								
**Family: Salpidae**																
*T*. *democratica*	-	-	25	10	43	3.6	-	23.8	88.4	86.6	-	44.7	67.9	66.6	83.1	23.4
*Brooksia rostrata*	-	-	75	90	13.8	2.1	-	8.6	11.6	13.4	-	3.7	32.7	33.3	16.9	29.8
**Total amount (ind./m**^**3**^**)**	**32.4**	**28.6**	**3.7**	**5.9**	**39.8**	**19.5**	**12.8**	**67.6**	**5.2**	**6**	**95.9**	**31.7**	**11.9**	**5.4**	**149.4**	**195.2**

### Pelagic tunicates and environmental variables

**Fig 5:**
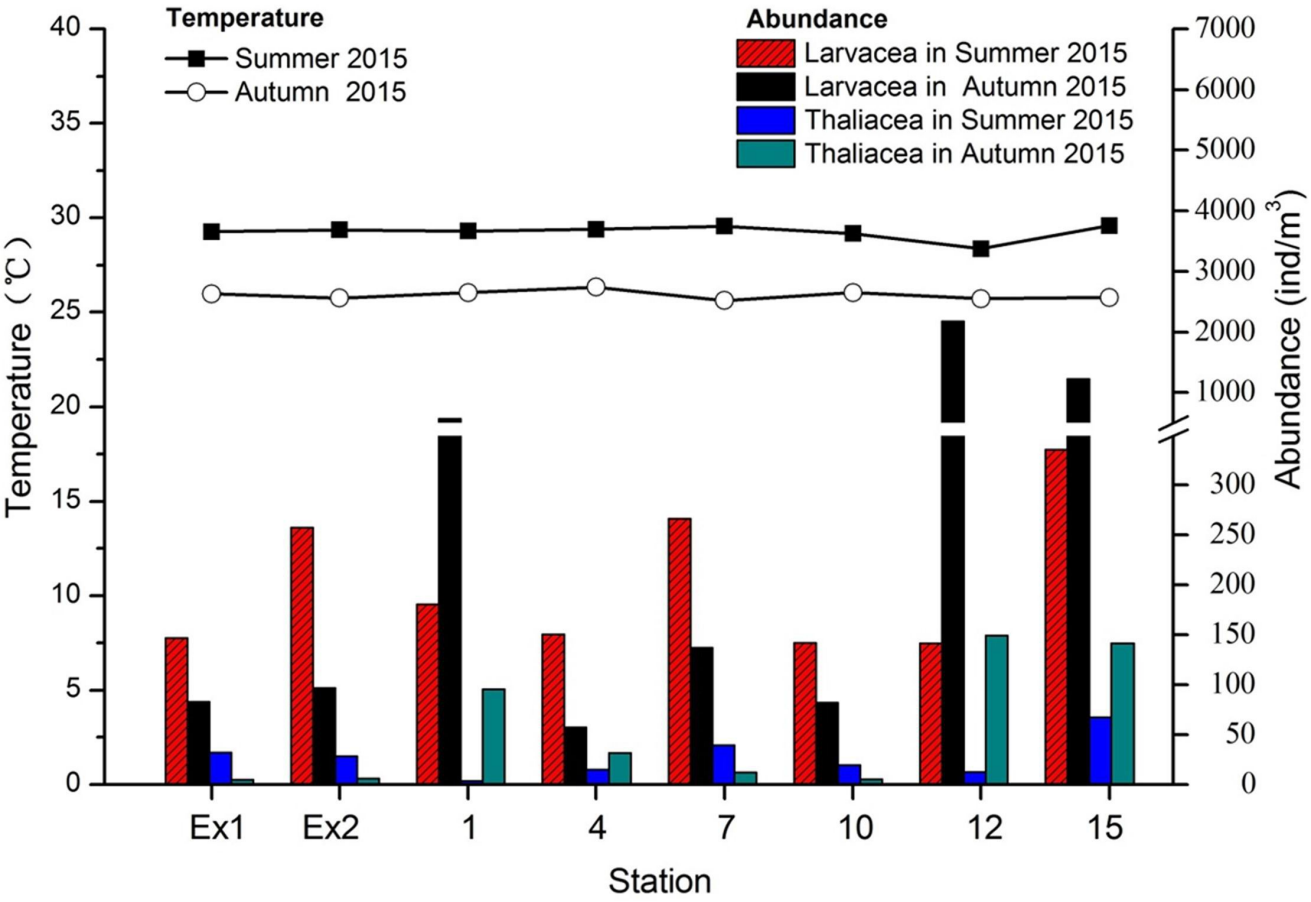
Total abundance of pelagic tunicates, and the total average temperature in the study area for every station.

**Fig 5** shows the total abundances of pelagic tunicates and total average temperature values in the study area during summer and autumn 2015 for every station. The highest values of tunicates were measured at stations 12, 15, and 1 during autumn. We used Pearson correlation to understand the influence of environmental factors on the abundances of pelagic tunicates and the results are shown in **Table 7**. Only Larvaceans and Thaliaceans were positively correlated. Temperature showed the unprecedented case of a negative correlation with the abundance of tunicates.

**Table 7:** Results of the Pearson correlation model analyzing the abundances of the two classes of organisms and environmental factors for the study regarding the summer and autumn comparison.

* *	Temperature	Salinity	Oikopleuridae	Thaliacea
**Temperature**	1			
**Salinity**	0.379758	1		
**Oikopleuridae**	-0.32877	-0.32256	1	
**Thaliacea**	-0.27087	-0.23149	0.900653	1

**Fig 6:**
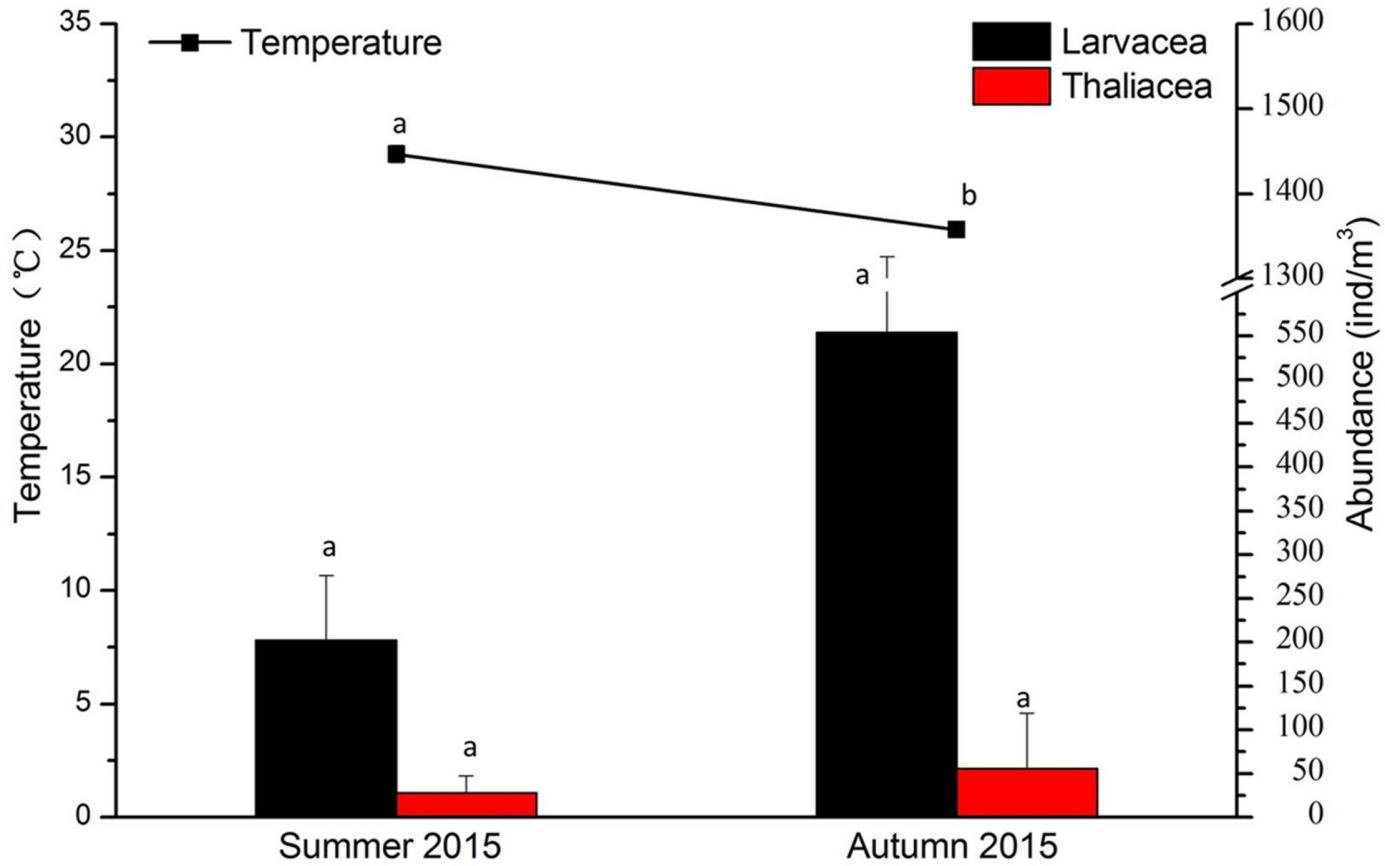
Changes in temperature and abundance of Thaliacea and Oikopleuridae for the two seasons surveyed (summer and autumn) (average values for seasons and the entire area). Different letters indicate significant differences (p<0.05).

**Fig 6** showed the results of our ANOVA statistical model. Temperature average values decreased in autumn as expected. Also, the abundance of Larvacea dropped that year as the statistical model showed. Surprisingly, larvaceans and thaliaceans were more abundant during the autumn season compared to the summer period.

## Discussion

The present study was focusing on the taxonomic composition, spatial distribution and abundance of appendicularians, salps, and doliolids in the waters around Kueishantao (Turtle Island) in north-eastern Taiwan in relation to environmental factors. We will discuss our results here within the three different subject matters we were studying.

### Surface collection *vs*. oblique tow collection in autumn 2015

There is a strong difference in results considering the abundances of organisms collected by the two different techniques. Larvaceans were more abundant than thaliaceans at all surveyed stations. Larvaceans prefer surface waters for their spawning [14] and thaliaceans tend to bloom in surface waters when the availability of food is high. Even though doliolids do not show a clear diel vertical migration [15]. They can live in different water depths according to Jellies Zone - *Dolioletta gegenbauri* Uljamin, 1884: Doliolid (http://jellieszone.com/pelagic-tunicates/nathistoc.bio.uci.edu/ - accessed on 2015/09). In another study [16] the different life stages showed up at different water depths. The surface collection method proved to be a more efficient way to collect pelagic tunicates compared to oblique tows, likely because tunicates occurred in the surface waters during night time when sampling took place. As shown in **Fig 2**, higher values of organism abundances were resulting from surface collections. The values at station EX1 and EX2 were quite similar for both techniques since the position of the two stations was right close to the island shores, and their shallowness (max. 20 meter water depth). The use of the oblique tow technique to collect pelagic tunicates aimed at collecting thaliaceans because they are known to migrate through the water column for their feeding and reproduction. The results of this sampling were significantly inferior to the ones obtained using surface collections. Oblique towing did not allow to collect the more numerous organisms present in the surface waters, explaining the lower values.

### Comparison between abundances and distribution of tunicates throughout a period of three autumns (2009, 2014, 2015)

The three seasons showed different results in tunicate abundances. Thaliacean abundances were low during the three years. As for larvaceans, the autumn of 2015 showed the highest and autumn 2014 the lowest abundances. **Fig 3** shows the abundance of pelagic tunicates for each station during the three surveyed years. The stations EX1 and EX2 showed the lowest values for larvaceans and thaliaceans on average. We hypothesized that, due to the presence of the hydrothermal vent plumes underwater which create a toxic environment for the organisms, the results showed a minimal presence of the same organisms. Pelagic tunicates are zooplankton organisms which as such are transported by water masses and currents. However, considering that pelagic tunicates show propulsion movements, we still expect that these species manage to avoid getting into the toxic water plume of the hydrothermal vents.

Our statistical analysis demonstrated the influence of temperature and salinity changes on the organisms throughout the study area. Salinity changes were not significantly different throughout the year (average 33 ppm). However, temperature played an important role for the organisms present in the area. In fact, the Pearson correlation and ANOVA test we carried out, both showed a negative correlation between larvacean abundance and temperature (**Fig 4**). Thaliaceans were not affected by any of the environmental variables measured in this study. *Oikopleura* (Larvacea) comprise of eurythermal and euryhaline species [17]. Franco and coworkers [18] in their study in the Yellow Sea showed that following a rise of temperature, the abundances of these organisms dropped significantly. This is exactly what we observed in the present study. In autumn 2014, the temperature values were the highest (compared to the other two autumns) and larvaceans values were the lowest (**Fig 5**). Larvaceans might not spawn when the water temperature is too warm. Pearson correlation also showed that there was a positive correlation between the two different classes of pelagic tunicates. Xu and Zhang [19] provided a model for determining optimal temperature and salinity showing its regulatory function for Appendicularia in the East China Sea. Nakamura [1] showed in 1998 that both larvaceans and thaliaceans can be found at high patches of abundance in different areas. In our research, they did not show competition for food. The environmental conditions seemed preferable for both classes.

### Comparison between abundances and distribution of organisms collected in summer 2015 and autumn 2015

Our third study focused on two seasons (summer and autumn). **Fig 6** shows the changes of abundances of organisms at all stations of the surveyed area during the two different seasons. Station EX1 and EX2 showed the lowest values on average. This may be caused, as mentioned above, by the presence of toxic waters from the hydrothermal vents located below. We tried again with statistical models, to identify any effect of environmental factors on the organisms. Unfortunately, we could not measure the chl-*a* values in our survey. This would have provided information for the understanding to what extend food might have played a role in the presence of pelagic tunicates in this particular environment. For example, the station number 4 also showed low values of pelagic tunicates but we could not find any reason for that. With appropriate chl-*a* data we would have been able to find answers. *Oikopleura* and thaliaceans generally had two times higher abundances in autumn than in summer. The results were similar to the second part of the study. Temperature was negatively correlated to the abundance of pelagic tunicates. Beyond a certain temperature, pelagic tunicates were not found at certain localities. In this study, the correlation between larvaceans and thaliaceans was high, as shown in the result table of the Pearson correlation (**Table 7**). As we described in the second part of the study, the two tunicate groups did not compete in the environment and they both spawned when suitable conditions appeared.

### Conclusions

Our survey had the objective to understand the community structures of pelagic tunicates in the waters of Kueishantao (Turtle Island), in Taiwan. Considering the limited number of species of pelagic tunicates worldwide, the fact that we could find an average of 10 different species in this area indicated an important biodiversity hot spot. The presence of toxic waters from hydrothermal vents showed an effect on the organisms, with lowest abundances at the stations located at the plume. The best technique to collect pelagic tunicates in the area was by surface sampling. We found that larvaceans were most abundant here. A reason for thaliaceans to have low concentrations during our study is explained by their ecology and reproductive behavior. Since salps show vertical migration, a survey from deeper waters could provide different results. For this it would be necessary to conduct a different sampling approach in the area, for instance by sampling from depth throughout the entire water column (we suggest from down to 200 m depth). This sampling should also consider the vertical and seasonal changes in temperature and salinity throughout the area.

## References

[1] Nakamura Y., 1998 Blooms of tunicates *Oikopleura* spp. and *Dolioletta gegenbauri* in the Seto Inland Sea, Japan, during summer. *Hydrobiologia*. 385(1–3): 183–192.

[2] Cristian A. V., Madin L. P., 2004 Zooplankton feeding ecology: clearance and ingestion rates of the salps *Thalia democratica*, *Cycosalpa affinis*, and *Salpa cylindrica* on naturally occurring particles in the Mid-Atlantic Bight. J. Plankton Res. 26(7): 827–833.

[3] Alldredge A. L., 2005 The contribution of discarded appendicularian houses to the flux of particulate organic carbon from oceanic surface waters In: *Response of marine ecosystems to global change: ecological impact of appendicularians*. Gorsky G., Youngbluth M. J., Deibel D. (eds). GB Scientific Publisher, Paris, 309–326.

[4] Alldredge A. L., Madin L. P., 1982 Pelagic Tunicates Unique Herbivores in the Marine Plankton. *Bioscience* 32(8): 655–663.

[5] Alldredge A. L., 1981 The Impact of appendicularian grazing on natural food concentrations *in situ*. *Limnol*. *Oceanogr*. 26 (2): 247–257.

[6] Madin L. P., Purcell J. E., Miler C. B., 1997 Abundance and grazing effects of *Cydosalpa bakeri* in the subarctic Pacific. Mar. Ecol.-Prog. Ser. 157:175–183.

[7] Kannathasan A., Ezhilarasan A., Sampathkumar P., Balamurugan K., 2012 Seasonal distribution of pelagic tunicates with influence of the environmental parameters in the Parangipettai, southeast coast of India. Pelagic Research Library. *Adv. Appl*. *Sci*. *Res*. 3(6): 3714–3721.

[8] Hwang J. S., Tu Y. Y., Tseng L. C., Fang L. S., Souissi S., Fang T. H. et al, 2004 Taxonomic composition and seasonal distribution of copepod assemblages from waters adjacent to nuclear power plant I and II in northern Taiwan. *J*. *Mar*. *Sci*. *and Technol*. 12 (5): 380–391.

[9] Kâ S, Hwang J. S., 2011 Mesozooplankton distribution and composition on the northeastern coast of Taiwan during autumn: effects of the Kuroshio Current and hydrothermal vents. *Zool*. *Stud*. 50(2): 155–163.

[10] Zhang J. B., Huang J. X., Lian G. S., 2003 Species composition and abundance distribution of Thaliacea in late autumn and early winter in the Nanwan Bay of Taiwan, China. *Bull*. *Mar*. *Sci*. 22 (6): 9–16.

[11] Franco P, Chen HJ, Hwang JS. 2016 Taxonomic composition and seasonal distribution of pelagic tunicates in the waters off nuclear power plants in northern Taiwan in relation to environmental conditions. *Zool*. *Stud*. MS: 20151018. 10.6620/ZS.2016.55-28 PMC6511830 31966173

[12] Chiu C. L., Song S. R., Hsieh Y. C., Chen C.-X., 2010 Volcanic characteristics of Kueishantao in northeast Taiwan and their implications. *Terr*. *Athmos*. *Ocean*. *Sci*. 21(3): 575–585.

[13] Chen C. T. A., Zeng Z., Kuo F. W., Yang T. F., Wang B. J. and Tu Y. Y., 2005 Tide-influenced acidic hydrothermal systems offshore NE Taiwan. *Chem*. *Geology*. 224: 69–81.

[14] Alldredge A. L., 1976a Field behavior and adaptive strategies of appendicularians (Chordata: Tunicata). *Limnol Oceanogr*., 21, 14–23.

[15] Gibson D. M., Paffenhöfer G. A., 2000 Feeding and growth rates of the doliolid, *Dolioletta gegenbauri* Uljanin (Tunicata, Thaliacea). *J*. *Plankton Res*., 22 (8): 1485–1500.

[16] Tew K. S., Lo W. T., 2005 Distribution of Thaliacea in SW Taiwan coastal waters in 1997, with special reference to *Doliolum denticulatum*, *Thalia democratica*, and *T*. *orientalis*. *Mar*. *Ecol*. *Prog*. *Ser*. 292: 181–193.

[17] Alldredge A. L., 1976b Appendicularians. *Scient*. *American* 235: 95–102.

[18] Franco P., Chen H. J., Liu G. X., 2013 Distribution and Abundance of Pelagic Tunicates in the North Yellow Sea. 10.1007/s11802-014-2376-0 ISSN 1672-5182. J. Ocean Univ. China (O*ceanic and Coastal Sea Research)* 2014 13 (5): 782–790.

[19] Xu Z. L., Zhang D., 2010 Yield-density model for determining optimal temperature and salinity for zooplankton: case studies with Appendicularia in the East China Sea. *Bull*.*Mar*. Sci. 86 (1): 149–164.

